# Calcium Hypophosphite: A New Active Ingredient for Biomimetic Oral Care

**DOI:** 10.3390/biomimetics11060403

**Published:** 2026-06-08

**Authors:** Joachim Enax, Pascal Fandrich, Erik Schulze zur Wiesche, Bennett T. Amaechi

**Affiliations:** 1 Research Department, Dr. Kurt Wolff GmbH & Co. KG, Johanneswerkstr. 34–36, 33611 Bielefeld, Germany; 2 Department of Comprehensive Dentistry, School of Dentistry, University of Texas Health San Antonio, 7703 Floyd Curl Drive, San Antonio, TX 78229-3900, USA

**Keywords:** biomimetics, calcium compounds, calcium hypophosphite, dental caries, dental enamel, dietary supplements, food preservatives, oral health, remineralization, toothpastes

## Abstract

Hypophosphites (also known as phosphinates) are the salts of hypophosphorous acid (also known as phosphinic acid), H_3_PO_2_. Various hypophosphite salts are known such as calcium hypophosphite (Ca(H_2_PO_2_)_2_) and sodium hypophosphite (Na(H_2_PO_2_)). Hypophosphites were proposed as a potential treatment for tuberculosis as early as the 1850s; however, they were found to be ineffective against this disease. Following this, there was a period of around 100 years during which no new studies on hypophosphites were published. Subsequent in vitro studies have shown that hypophosphites can be used as potential antibacterial food preservatives. Currently, calcium hypophosphite is used in commercial food supplements for children, as this compound is a suitable source of calcium ions. It also has other advantageous properties, including exceptionally high water solubility (154 g/L at 25 °C), a neutral taste, a high mass fraction of calcium per molecule (23.6%), and an excellent safety profile. Recent studies have shown its potential as an active ingredient in the field of oral care. Since biological mechanisms such as tooth and bone formation and natural remineralization due to saliva rely on calcium ions, calcium hypophosphite can be regarded as a biomimetic agent. Upon contact with phosphate from saliva, calcium hypophosphite forms hydroxyapatite; this imitation of physiological mineralization and crystallization processes in the human body further underlines its biomimetic character. This review summarizes and discusses the available literature on hypophosphites in human health and related fields.

## 1. Introduction

### 1.1. Chemical Background of Hypophosphites

Hypophosphites (also known as phosphinates) are the salts of hypophosphorous acid (also known as phosphinic acid), H_3_PO_2_ [1,2,3]. Various hypophosphite salts are known, including calcium hypophosphite (Ca(H_2_PO_2_)_2_), manganese hypophosphite (Mn(H_2_PO_2_)_2_), sodium hypophosphite (Na(H_2_PO_2_)), and potassium hypophosphite (K(H_2_PO_2_)) [4,5]. The oxidation state of phosphorus in the hypophosphite molecule is +I. The p*K*_a_ of hypophosphorous acid is 1.23 [1], i.e., it is a moderately strong acid and thus almost exclusively present in a dissociated form in solution at neutral pH.

**Figure 1:**
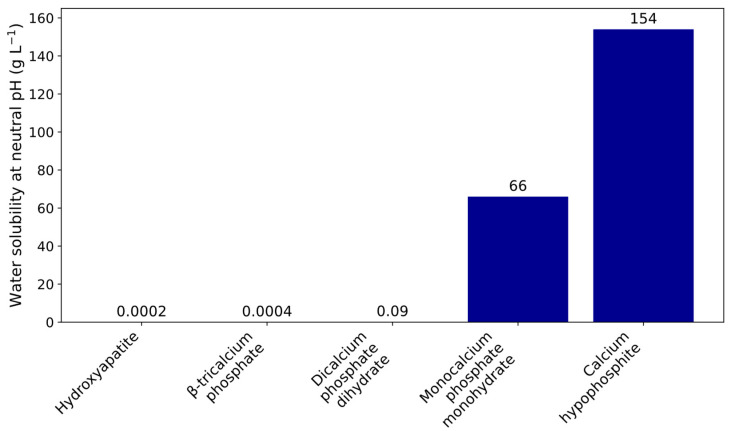
Comparison of water solubility of calcium hypophosphite (calcium phosphinate), Ca(H_2_PO_2_)_2_ [7], and selected calcium phosphates (hydroxyapatite, Ca_5_(PO_4_)_3_(OH) [8]; β-tricalcium phosphate, β-Ca_3_(PO_4_)_2_ [8]; dicalcium phosphate dihydrate (brushite), CaHPO_4_ ∙ 2 H_2_O [8]; monocalcium phosphate monohydrate, Ca(H_2_PO_4_)_2_ ∙ H_2_O [8]) at neutral pH. Note that all calcium phosphates are soluble under acidic conditions.

In general, all hypophosphite salts are highly soluble in water [1,6,7]. Compared to other calcium-phosphorus compounds, such as calcium phosphates, calcium hypophosphite has a much higher water solubility at neutral pH (154 g L^−1^ at 25 °C) (Figure 1).

The hypophosphite anion exhibits antimicrobial activity against certain microorganisms [4,9,10,11]. Thermal decomposition of hypophosphites is very unlikely as it only occurs at elevated temperatures (*T* > 300 °C) [2,12].

As a calcium salt, calcium hypophosphite can serve as a source of calcium ions, making it a suitable ingredient for use in food supplements for individuals with calcium deficiencies [2,6]. Calcium hypophosphite offers several advantages as a food supplement, including high water solubility (154 g L^−1^ at 25 °C; see also Figure 1), a neutral taste, a high molar ratio of calcium per molecule (23.6%; see also Table 3), and a high safety profile [4,6,13].

Interestingly, hypophosphites were proposed as a strategy to treat tuberculosis as early as the 1850s [4]. However, their efficacy against this disease has not been proven [4]. In the 1980s, hypophosphites were introduced to food technology as an antibotulinal agent and later for various other purposes, including use as antioxidants and as agents with efficacy against certain foodborne bacteria [4,9,10,14].

Calcium hypophosphite (CAS number: 7789-79-9; *M* = 170.1 g mol^−1^) has many synonyms, such as calcium phosphinate, phosphinic acid calcium salt, calcii phosphinas, calcium phosphinicum, and calcium hypophosphorosum [2]. The two most commonly used names in the literature are (A) calcium hypophosphite [4,6,7,13] and (B) calcium phosphinate (according to the International Nomenclature of Cosmetic Ingredients (INCI) [15]).

**Figure 2:**
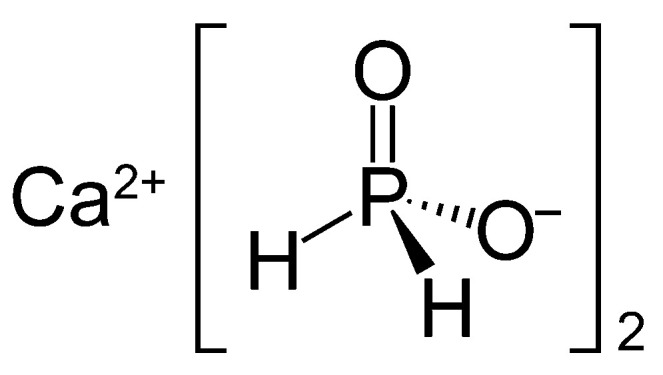
Chemical configuration of calcium hypophosphite (calcium phosphinate). The H_2_PO_2_^−^ anion has a tetrahedral structure [1]. Note that the calcium ion can be substituted by other metal ions such as sodium [sodium hypophosphite, Na(H_2_PO_2_)] or manganese [manganese hypophosphite, Mn(H_2_PO_2_)_2_].

The chemical structure of calcium hypophosphite is depicted in Figure 2.

### 1.2. Calcium Hypophosphite and Biomimetic Oral Care

As described above, calcium hypophosphite has many characteristics that make it a versatile ingredient for various applications in human health as well as related fields (e.g., food technology). Since biological mechanisms, such as the formation of the mineral phase during tooth development [16], natural remineralization of teeth due to saliva [17], and biomineralization processes in bone [18], rely on calcium ions, calcium hypophosphite—as an efficient source of calcium ions—can be regarded as a biomimetic agent. In natural biomineralization processes, calcium ions in the presence of phosphate ions first form amorphous calcium phosphate (ACP), which is then transformed into dicalcium phosphate dihydrate (DCPD), followed by octacalcium phosphate (OCP), and can finally transform into hydroxyapatite (HAP) [17]. This calcium-ion-based approach using calcium hypophosphite could represent an additional biomimetic strategy in oral care. Typically, biomimetic oral care relies on synthetic hydroxyapatite crystallites, which have long been established as biomimetic agents that imitate the structure and composition of natural enamel crystallites [19]. In contrast, the use of calcium hypophosphite addresses a different biomimetic aspect, as it provides freely available calcium ions, whereas in hydroxyapatite, these ions are fixed within the apatite lattice.

### 1.3. Aim of the Review

The aim of this narrative review is to summarize and discuss the available literature on hypophosphite salts in fields related to human health and to identify potential areas for future research.

## 2. Materials and Methods

The scientific literature search was conducted using various scientific databases, i.e., SciFinder, PubMed, and Google Scholar (including standard Google search), with results up to 27 April 2026 being considered.

The search terms used were as follows: “hypophosphite”, “phosphinate”, “calcium hypophosphite”, “calcium phosphinate”, “phosphinic acid calcium salt”, “calcii phosphinas”, “calcium phosphinicum”, and “calcium hypophosphorosum”. The synonyms of calcium hypophosphites were taken from ref. [2]. Afterwards, results describing calcium hypophosphite for technical applications (e.g., as a flame retardant [20]) were excluded by the authors. The search was limited to scientific articles and books in English and German, and patent applications were excluded. Additionally, a handbook of inorganic chemistry was used [1].

In addition to the literature search, an online product database search was conducted using Mintel (Mintel Group Ltd. London, UK) to identify commercial products containing calcium hypophosphite. All products that listed “calcium hypophosphite” as an ingredient were included. The Mintel database includes products available globally across the following categories: “beauty and personal care”, “household”, “pet”, and “health”. The Mintel search was completed on 27 April 2026.

## 3. Results and Discussion

### 3.1. Number of Results

After applying the inclusion and exclusion criteria, the literature search resulted in 13 relevant publications on hypophosphites, including calcium hypophosphite and sodium hypophosphite (Table 1). The details will be discussed in the corresponding sections. The product search resulted in 44 products containing calcium hypophosphite (see Section 3.2).

In the following sections, the potential use of hypophosphites as food supplements (Section 3.2), as antibacterial food preservatives (Section 3.3), and as ingredients in oral care (Section 3.4), as well as other applications (Section 3.5) will be presented and discussed.

**Table 1:** Overview of the literature on hypophosphites (phosphinates) including calcium hypophosphite (calcium phosphinate).

Type of Article	Topic	Number of Publications	Reference(s)
Book chapters	Chemistry of hypophosphite	2	[1,3]
Review article	Use of hypophosphites in food applications	1	[4]
Original research articles	Remineralization of initial caries lesions using calcium hypophosphite (in vitro)	1	[21]
	Use of calcium hypophosphite as calcium supplement (*animal model*)	1	[6]
	Use of sodium hypophosphite as antibacterial food preservative (in vitro)	3	[9,10,14]
	Use of sodium hypophosphite against formate-producing bacteria (in vitro)	1	[11]
Conference abstracts	Remineralization of initial caries lesions using calcium hypophosphite (in vitro) Occlusion of open dentin tubules by mineral precipitates formed from the reaction of calcium hypophosphite with phosphate from artificial saliva (in vitro)	2	[22] (for full publication see [21]) [23]
Scientific opinions	Use of calcium hypophosphite as calcium supplement Evaluation of the health aspects of hypophosphites as food ingredients	1 1	[2] [13]
Hypothesis	Use of calcium hypophosphite for treating obesity in humans	1	[7]

### 3.2. Calcium Hypophosphite (Calcium Phosphinate) as Food Supplement

Calcium is an essential component of the mineral phase of human teeth and bones, which is present as hydroxyapatite crystallites, Ca_5_(PO_4_)_3_(OH) [18]. The calcium content in tooth enamel is 36.5%, 35.1% in dentin, and 34.8% in bone [18], i.e., calcium ions represent an important constituent of the mineral phase of human teeth and bones.

Additionally, calcium is essential for the normal function of cells including blood, muscle, heart, and nerve cells [24]. Although calcium is essential for humans, calcium deficiency is quite common worldwide, even in high-income countries [25,26].

Calcium hypophosphite can be used as calcium source for use in food supplements for several reasons: it has high water solubility, a neutral taste, a high content of calcium per molecule (23.6%), and a high safety profile [4,6,13].

Commercially available food supplement products for humans containing calcium hypophosphite are available. The global product search for calcium hypophosphite using the Mintel database yielded 44 results (Table 2). The products that were found were released in the period from July 2002 to November 2025. In all cases, calcium hypophosphite was used as a source of calcium ions in food supplements, such as multivitamin syrups and cod liver oil emulsions. It is worth mentioning that the majority of the products identified in the product search were specifically intended for children, further confirming the safety of calcium hypophosphite.

The concentration of calcium hypophosphite in the products ranges from 0.3% to 2.76%, which was calculated from the information on the respective product labels. Interestingly, the highest number of products containing calcium hypophosphite were found in the Asia-Pacific region (Table 2).

While there are other calcium sources, such as calcium carbonate (CaCO_3_) and calcium citrate (Ca_3_(C_6_H_5_O_7_)_2_) [26], that are more commonly used in food supplements than calcium hypophosphite, the availability of products containing calcium hypophosphite highlights its ability to act as a suitable calcium source for humans (Table 2).

After calculating the mass fraction of calcium to the respective calcium compounds, it was found that the mass fraction for calcium hypophosphite is similar to that of calcium citrate and lower than that for calcium carbonate (Table 3). It is worth noting that the water solubility of calcium hypophosphite is significantly higher than that of calcium citrate and calcium carbonate (Table 3).

**Table 3:** Comparison of calculated mass fraction of calcium to calcium compound and water solubility of calcium hypophosphite (calcium phosphinate) compared to calcium carbonate and calcium citrate, the two most common calcium compounds in calcium supplements [26].

Calcium Compound	Calculated Mass Fraction of Calcium-to-Calcium Compound (%)	Water Solubility at Neutral pH (g L^−1^)	Reference
Calcium hypophosphite Ca(H_2_PO_2_)_2_	23.6	154	[7]
Calcium citrate Ca_3_(C_6_H_5_O_7_)_2_	24.1	0.95	[27]
Calcium carbonate CaCO_3_	40.0	Practically insoluble in water	[28]

In contrast to calcium, the bioavailability of phosphorus from hypophosphites has been found to be not significant (which can be explained by the stability of the hypophosphite anion under in vivo conditions) [4,13,29].

Notably, calcium hypophosphite is used not only in humans but also in animals, administered as infusions for horses, goats, cattle, pigs, and dogs to treat calcium deficiency [30].

### 3.3. Use of Hypophosphites (Phosphinates) as Antibacterial Food Preservative

In vitro studies have shown that hypophosphites, specifically sodium hypophosphite, have the potential to be utilized as an antibacterial food preservative [4,9,10]. Rhodehamel and Pierson conducted two in vitro studies analyzing the efficacy of sodium hypophosphite in inhibiting both Gram-negative and Gram-positive bacteria [9,10].

Sodium hypophosphite has been shown to reduce the growth of certain microorganisms, including *Salmonella typhimurium* and *Vibrio parahaemolyticus*. However, it has no effect on the growth of other microorganisms, such as *Campylobacter jejuni* (strain Smith) and *Bacillus cereus* (Table 4) [9,10].

The antibacterial effect of sodium hypophosphite is primarily attributed to the hypophosphite anion, H_2_PO_2_^−^ [9]. Therefore, similar antibacterial effects can be anticipated with other hypophosphite salts, such as calcium hypophosphite.

Hypophosphites have the advantage of exhibiting antibacterial properties even under near-neutral pH conditions, making them potential preservative agents in food products with low acid levels [4].

To the best of the authors’ knowledge, no recent follow-up studies in this research field have been conducted. Therefore, further research is necessary to analyze the potential and suitability of hypophosphites as antibacterial food preservatives.

### 3.4. Use of Calcium Hypophosphite (Calcium Phosphinate) in Oral Care

Caries is a disease caused by cariogenic bacteria that metabolize sugars into acids, which can attack the teeth [31]. Therefore, different strategies are employed for caries prevention, including remineralization and the use of antibacterial approaches [32,33].

Calcium-containing ingredients are used as anti-caries agents in toothpastes. Examples of calcium-containing ingredients in oral care include various calcium phosphates, e.g., amorphous calcium phosphate in complex with casein phosphopeptide (CPP-ACP) and hydroxyapatite (HAP), as well as calcium lactate and calcium carbonate.

Calcium hypophosphite is highly soluble in water, i.e., it readily provides calcium ions, making it a suitable active ingredient for oral care products such as toothpastes and mouthwashes.

It should be noted that the ability to provide calcium ions is not unique to calcium hypophosphite, as other soluble calcium salts may also increase the calcium ion concentration. Calcium hypophosphite is distinguished by a specific combination of properties relevant for oral care applications, including high water solubility, a neutral taste, a favorable safety profile, and established use as a calcium source in food supplements. In addition, the reported antibacterial effects of the hypophosphite anion against selected microorganisms in non-oral model systems provide a promising basis for future studies investigating the effects of calcium hypophosphite on microorganisms relevant to oral health, including oral bacteria.

**Figure 3:**
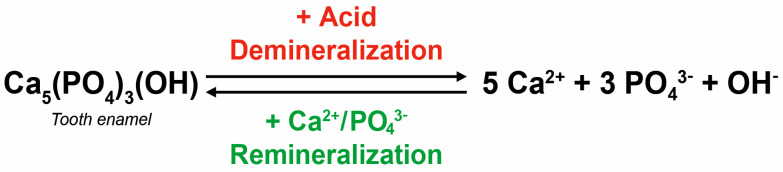
Schematic depiction of the dynamic equilibrium between demineralization and remineralization of tooth enamel. Acidic conditions (caries, erosion) lead to demineralization of enamel, while the presence of calcium and phosphate ions promotes remineralization (i.e., hydroxyapatite formation). Saliva naturally contains calcium and phosphate ions. The remineralization process can be further enhanced by using oral care products that contain calcium and/or phosphate (in both particulate and ionic forms).

As the demineralization and remineralization of teeth are in constant dynamic equilibrium, the provision of calcium ions can shift the equilibrium towards remineralization (i.e., hydroxyapatite formation), in accordance with Le Chatelier’s principle (Figure 3).

Calcium hypophosphite provides calcium ions essential for tooth remineralization, thereby mimicking the natural remineralization process [17].

A recent in vitro study has demonstrated a positive correlation between the concentration of water-soluble calcium compounds in oral care products and tooth remineralization [34].

Another study by Shaw et al. supports this finding, showing that plaque in caries-free children has a significantly higher calcium concentration than plaque in children with high caries activity [35]. Significantly higher calcium concentrations were found in both posterior and anterior plaque of caries-free children (3.57 µg mg^−1^ and 11.55 µg mg^−1^) compared to caries-active children (1.63 µg mg^−1^ and 2.57 µg mg^−1^) [35]. Additionally, the presence of calcium ions (and phosphate ions) is known to reduce the demineralizing effect of acids on teeth [36].

Calcium hypophosphite can be added to hydroxyapatite-based oral care products to provide long-term availability of calcium ions for remineralization. Its high water solubility allows the release of calcium ions during tooth brushing, while hydroxyapatite releases calcium ions at the tooth surface under acidic conditions, such as in cariogenic biofilms [37]. Therefore, regarding tooth remineralization, synergistic effects with other calcium phosphates, such as hydroxyapatite [19], can be expected.

A recent in vitro study on toothpastes demonstrated that calcium hypophosphite effectively remineralizes initial caries lesions induced in a microbial caries model (Table 5) [21]. The remineralization efficacy of a toothpaste containing 1% calcium hypophosphite was significantly higher than that of a toothpaste containing sodium fluoride (1450 ppm fluoride). It was also significantly more effective than a toothpaste with 20% hydroxyapatite. The most effective remineralization was achieved with a combination of 1% calcium hypophosphite and 20% hydroxyapatite (Table 5). Although not analyzed in this study, from a chemical perspective, a higher concentration of calcium hypophosphite may be even more efficient in remineralization because it could induce a higher calcium concentration, shifting the remineralization/demineralization equilibrium towards remineralization, i.e., hydroxyapatite formation (Figure 3).

**Figure 4:**
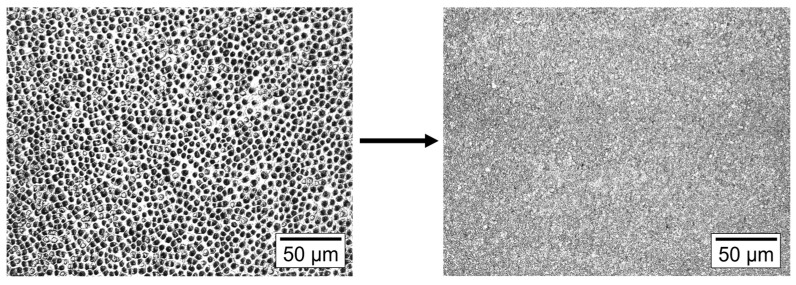
Representative microscopic image illustrating the occlusion of open dentin tubules by mineral precipitates formed in artificial saliva after treatment with calcium hypophosphite. The mineral precipitates consisted of hydroxyapatite.

A recent in vitro study investigated the effects of calcium hypophosphite on dentin tubules in the presence of artificial saliva [23]. Open dentin tubules are known to be the main cause for sensitive teeth (dentin hypersensitivity) [38]. Calcium hypophosphite spontaneously forms a mineral precipitate consisting of hydroxyapatite when in contact with phosphate from saliva [23]. Laser scanning microscopy analyses showed that this mineral precipitate efficiently occludes open dentin tubules (Figure 4) [23]. Unlike particles with a fixed particle size distribution, these in situ-formed precipitates can lead to tailored occlusion of dentin tubules.

Future studies could further characterize the mineral precipitates formed by calcium hypophosphite in saliva-like environments with regard to additional parameters, including crystallite morphology and size.

### 3.5. Further Potential Uses of Hypophosphites (Phosphinates)

In addition to its potential use as a food supplement, antibacterial food preservative, and oral care ingredient, other applications for hypophosphites have been proposed.

A recent in vitro study has shown that sodium hypophosphite can reduce both the growth and formic acid production of bacteria such as *Gardnerella vaginalis 315-A*, *Amygdalobacter nucleatus*, *Mobiluncus mulieris*, and *Sneathia vaginalis*, which are associated with bacterial vaginosis [11].

Furthermore, there is a hypothesis that calcium or magnesium hypophosphite could be used in the treatment of obesity in humans [7]. Hypophosphites have also been described in the literature as having potential uses in the food industry as antioxidants, stabilizers, meat pickling accelerators, vegetable protein flow inducers, and antibotulinal agents [4].

Hypophosphites have a significant advantage in food technology due to their ability to inhibit bacteria even at neutral pH values. In contrast, other preservatives may show reduced antibacterial efficacy as the pH value decreases [4].

The United States Food and Drug Administration (FDA)’s Food Additive Status List categorizes sodium hypophosphite as an emulsifying and stabilizing agent and manganese hypophosphite as a dietary supplement and nutrient [5].

### 3.6. Safety of Hypophsophites (Phosphinates)

Hypophosphite salts easily dissolve in water, dissociating into metal cations (e.g., calcium or sodium) and hypophosphite anions. In the case of calcium hypophosphite, calcium cations and hypophosphite anions are formed when dissolved in water:

Ca(H_2_PO_2_)_2_ (s) → Ca^2+^ (aq) + 2 H_2_PO_2_^−^ (aq)

Therefore, in safety evaluations, both ions can be considered separately. In the case of calcium hypophosphite and sodium hypophosphite, the cations (Ca^2+^ and Na^+^) are also naturally present in the human body, making them safe. The hypophosphite anion is rapidly excreted unchanged in the urine after administration of hypophosphite salts [13,29].

The safety of hypophosphites is underscored by the fact that the calcium hypophosphite is used as a calcium source in food supplements and is also used in children (for details, see Section 3.2). Sodium hypophosphite and manganese hypophosphite are listed as food additives by the FDA, and have the status of being “Generally Recognized as Safe” (GRAS) [5]. In addition, calcium hypophosphite and potassium hypophosphite are also classified as GRAS substances by the FDA [39].

A report from the Federation of American Societies for Experimental Biology evaluating the health aspects of hypophosphites as food ingredients concluded that [13]:

“There is no evidence in the available information on manganous, calcium, potassium or sodium hypophosphite that demonstrated, or suggests reasonable grounds to suspect, a hazard to the public when they are used in the manner now practiced and at the levels that are now current or that might reasonably be expected in the future.”

## 4. Limitations

This review article has some limitations. Firstly, there is a limited number of studies available on hypophosphites including calcium hypophosphite (see also Table 1), and some of them are relatively old and some are preliminary studies. Thus, performing further research is important to confirm existing positive effects and to expand the scope to include other areas. Recently, the potential of calcium hypophosphite in the field of preventive oral health care has been explored and calcium hypophosphite has been shown to be a suitable active ingredient for oral care, e.g., for the remineralization of initial caries lesions [21].

Secondly, this review may have missed commercial products containing calcium hypophosphite because the Mintel database does not cover all product types, including various food products. Additionally, some of the products found in the Mintel database, particularly the older ones, may no longer be commercially available.

## 5. Outlook and Future Research

Future research on calcium hypophosphite is promising due to the positive results from preliminary studies in various fields, including food technology. Calcium hypophosphite is already used as a calcium source in commercially available food supplements, and its chemical properties, such as high water solubility and antibacterial effects, combined with a high safety profile, make it a promising ingredient for further research. The modes of action of hypophosphites, such as their antibacterial effects as food preservatives, require further research to fully understand the mechanisms and identify potential applications. Additionally, it is worth exploring potential synergistic effects of calcium hypophosphite with other active ingredients. The synergistic effects of calcium hypophosphite and hydroxyapatite in the remineralization of initial caries lesions have already been demonstrated [21].

## 6. Conclusions

Hypophosphites (also known as phosphinates), which include calcium hypophosphite (Ca(H_2_PO_2_)_2_) and sodium hypophosphite (Na(H_2_PO_2_)), are versatile compounds with potential applications in human health and related fields, including use as food supplements, for food preservation, and as active ingredients in oral care.

The high water solubility of calcium hypophosphite (154 g L^−1^) makes it a significant source of calcium ions that, when used in food additives, could counteract calcium deficiency.

The hypophosphite anion, H_2_PO_2_^−^, exhibits antibacterial properties against foodborne microorganisms.

Since biological processes such as tooth development, bone formation, and natural remineralization due to saliva depend on calcium ions, calcium hypophosphite can be considered a biomimetic agent. In the presence of phosphate from saliva, calcium hypophosphite forms hydroxyapatite; this replication of physiological mineralization and crystallization processes in the human body further highlights its biomimetic properties.

The availability of commercially available food supplements enriched with calcium hypophosphite, such as multivitamin syrups and cod liver oil emulsions, further demonstrates the safety of this compound. Additionally, various hypophosphite salts including calcium hypophosphite and sodium hypophosphites have been classified as “Generally Recognized as Safe (GRAS)” by the FDA.

## Figures and Tables

**Table 2:** Number of commercial products found in the Mintel database containing calcium hypophosphite (calcium phosphinate) as an ingredient. These products were released in the period from July 2002 to April 2026. It is worth noting that all identified products fell under the category of food supplements, with calcium hypophosphite being used as a source of calcium.

Region	Number of Products with Calcium Hypophosphite
Asia-Pacific	29
Middle East and Africa	9
Latin America	4
Europe	1
North America	1

**Table 4:** Effect of sodium hypophosphite (sodium phosphinate) on the growth of selected pathogenic and spoilage-causing microorganisms in food (taken from refs. [9,10]).

Microorganism	Effect on Growth
*Campylobacter jejuni*, strain H-840	−/+
*Campylobacter jejuni*, strain Smith	−
*Enterobacter aerogenes*	+
*Escherichia coli*	++
*Pseudomonas fluorescens*	−/+
*Salmonella typhimurium*	+++
*Vibrio parahaemolyticus*	+++/++++
*Yersinia enterocolitica*	++
*Bacillus cereus*	−
*Staphylococcus aureus*	+
*Clostridium perfringens*	++
*Clostridium botulinum*, strain 62A	+/++
*Clostridium botulinum*, strain 52A	+/++
*Clostridium botulinum*, strain Lamanna B	++

−: no effect (0–10% inhibition); +: slightly effective (11–30% inhibition); ++: moderately effective (31–60% inhibition); +++: highly effective (>60% inhibition); ++++: complete inhibition (no significant growth observed).

**Table 5:** Efficacy of calcium hypophosphite (calcium phosphinate) in remineralizing initial caries in vitro. Remineralization was quantified as the change in surface microhardness (SMH) of each sample measured before and after toothpaste treatment and expressed as percent remineralization (%Rem). For more information, see Amaechi et al. [21].

Toothpaste	Mean Percentage Remineralization (±Standard Deviation)
20% hydroxyapatite + 1% calcium hypophosphite	89.7 ± 3.3
1% calcium hypophosphite	75.4 ± 5.5
20% hydroxyapatite	62.4 ± 4.8
Sodium fluoride (1450 ppm fluoride)	60.3 ± 7.8

## Data Availability

No new data were created or analyzed in this study. Data sharing is not applicable to this article.

## References

[1] Holleman A.F., Wiberg E. (2016). Anorganische Chemie.

[2] European Food Safety Authority (2009). Scientific opinion of the panel on food additives and nutrient sources added to food on calcium phosphinate as a source of calcium added for nutritional purposes to food supplements following a request from the European Commission. EFSA J..

[3] Anselmino O., Gilg E. (1926). Calcium hypophosphorosum. Kommentar zum Deutschen Arzneibuch 6. Ausgabe 1926.

[4] Rhodehamel E.J., Pierson M.D., Leifer A.M. (1990). Hypophosphite: A Review. J. Food Prot..

[5] The United States Food and Drug Administration (FDA) Food Additive Status List. https://www.fda.gov/food/food-additives-petitions/food-additive-status-list.

[6] Meyer A.E., Greenberg J. (1949). Value of calcium hypophosphite and other calcium compounds as calcium supplements in calcium-low diets. Proc. Soc. Exp. Biol. Med..

[7] Robertson D.S. (2006). Magnesium or calcium hypophosphite could be a treatment for obesity in humans. Med. Hypotheses.

[8] Schamel M. (2016). Novel Dual Setting Approaches for Mechanically Reinforced Mineral Biocements. Ph.D. Thesis.

[9] Rhodehamel E.J., Pierson M.D. (1990). Sodium hypophosphite inhibition of the growth of selected gram negative foodborne pathogenic and spoilage bacteria. J. Food Prot..

[10] Rhodehamel E.J., Pierson M.D. (1990). Sodium hypophosphite inhibition of the growth of selected gram-positive foodborne pathogenic bacteria. Int. J. Food Microbiol..

[11] Lee E.M., Srinivasan S., Purvine S.O., Fiedler T.L., Leiser O.P., Proll S.C., Minot S.S., Djukovic D., Raftery D., Johnston C. (2025). Syntrophic bacterial and host-microbe interactions in bacterial vaginosis. ISME J..

[12] Haynes W.M. (2016). CRC Handbook of Chemistry and Physics.

[13] Federation of American Societies for Experimental Biology (1977). Evaluation of the Health Aspects of Hypophosphites as Food Ingredients.

[14] Leifer A.M. (1983). Sodium Hypophosphite Inhibition of *Clostridium botulinum* in Pasteurized Comminuted Pork. Master’s Thesis.

[15] CosIng—Cosmetics Ingredients/Calcium Phosphinate. https://ec.europa.eu/growth/tools-databases/cosing/details/105479.

[16] Lacruz R.S., Habelitz S., Wright J.T., Paine M.L. (2017). Dental enamel formation and implications for oral health and disease. Physiol. Rev..

[17] Enax J., Fandrich P., Schulze zur Wiesche E., Epple M. (2024). The remineralization of enamel from saliva: A chemical perspective. Dent. J..

[18] Dorozhkin S.V., Epple M. (2002). Biological and medical significance of calcium phosphates. Angew. Chem. Int. Ed..

[19] Pawinska M., Paszynska E., Amaechi B.T., Meyer F., Enax J., Limeback H. (2024). Clinical evidence of caries prevention by hydroxyapatite: An updated systematic review and meta-analysis. J. Dent..

[20] Savas Atabek L., Tayfun U., Hancer M., Dogan M. (2019). The flame-retardant effect of calcium hypophosphite in various thermoplastic polymers. Fire Mater..

[21] Amaechi B.T., Vohra R., Abdollahi S., Yang K., Obiefuna A.C., Schulze zur Wiesche E., Enax J. (2026). Remineralization of early caries lesions by calcium hypophosphite in vitro: A surface microhardness study. BDJ Open.

[22] Amaechi B.T., Yang K., Fandrich P., Schulze zur Wiesche E., Enax J. (2025). Oral Presentation: Efficacy of Calcium Hypophosphite in Remineralizing Initial Caries In Vitro: IADR/PER General Session & Exhibition, Barcelona, Spain, June 27, 2025.

[23] Fandrich P., Hollmann B., Schulze zur Wiesche E., Enax J. (2025). Poster Presentation: Effects of Calcium Hypophosphite on Human Teeth In Vitro: IADR/PER General Session & Exhibition, Barcelona, Spain, June 28, 2025.

[24] Harvard, T.H. Chan School of Public Health The Nutrition Source: Calcium. https://www.hsph.harvard.edu/nutritionsource/calcium/.

[25] Shlisky J., Mandlik R., Askari S., Abrams S., Belizan J.M., Bourassa M.W., Cormick G., Driller-Colangelo A., Gomes F., Khadilkar A. (2022). Calcium deficiency worldwide: Prevalence of inadequate intakes and associated health outcomes. Ann. N. Y. Acad. Sci..

[26] Straub D.A. (2007). Calcium supplementation in clinical practice: A review of forms, doses, and indications. Nutr. Clin. Pract..

[27] Calcium Citrate. https://www.chemeurope.com/en/encyclopedia/Calcium_citrate.html.

[28] Calcium Carbonate. https://pubchem.ncbi.nlm.nih.gov/compound/Calcium-Carbonate.

[29] Braun U., Jehle W. (2007). The effect of intravenous magnesium hypophosphite in calcium borogluconate solution on the serum concentration of inorganic phosphorus in healthy cows. Vet. J..

[30] EU Veterinary Medicines. https://medicines.health.europa.eu/veterinary/en/search-medicines?keys=%22calcium%20hypophosphite%22.

[31] Fejerskov O., Kidd E. (2009). Dental Caries: The Disease and Its Clinical Management.

[32] ten Cate J.M. (2009). The need for antibacterial approaches to improve caries control. Adv. Dent. Res..

[33] van Loveren C. (2013). Toothpastes.

[34] Fernando J.R., Walker G.D., Park T.K.-S., Shen P., Yuan Y., Reynolds C., Reynolds E.C. (2022). Comparison of calcium-based technologies to remineralise enamel subsurface lesions using microradiography and microhardness. Sci. Rep..

[35] Shaw L., Murray J.J., Burchell C.K., Best J.S. (1983). Calcium and phosphorus content of plaque and saliva in relation to dental caries. Caries Res..

[36] Dawes C. (2003). What is the critical pH and why does a tooth dissolve in acid?. J. Can. Dent. Assoc..

[37] Cieplik F., Rupp C.M., Hirsch S., Muehler D., Enax J., Meyer F., Hiller K.-A., Buchalla W. (2020). Ca^2+^ release and buffering effects of synthetic hydroxyapatite following bacterial acid challenge. BMC Oral Health.

[38] Gillam D.G. (2015). Dentine Hypersensitivity: Advances in Diagnosis, Management, and Treatment.

[39] The United States Food and Drug Administration (FDA), Select Committee on GRAS Substances (SCOGS). https://www.cfsanappsexternal.fda.gov/scripts/fdcc/?set=SCOGS&sort=Sortsubstance&order=ASC&startrow=1&type=basic&search=hypophosphite.

